# The water-soluble fraction of extracellular polymeric substances from a resource recovery demonstration plant: characterization and potential application as an adhesive

**DOI:** 10.3389/fmicb.2024.1331120

**Published:** 2024-02-26

**Authors:** Le Min Chen, Özlem Erol, Young Hae Choi, Mario Pronk, Mark van Loosdrecht, Yuemei Lin

**Affiliations:** ^1^Department of Biotechnology, Delft University of Technology, Delft, Netherlands; ^2^Natural Products Laboratory, Institute of Biology, Leiden University, Leiden, Netherlands; ^3^Royal HaskoningDHV, Amersfoort, Netherlands

**Keywords:** extracellular polymeric substances, aerobic granular sludge, adhesive, resource recovery, shear strength

## Abstract

Currently, there is a growing interest in transforming wastewater treatment plants (WWTPs) into resource recovery plants. Microorganisms in aerobic granular sludge produce extracellular polymeric substances (EPS), which are considered sustainable resources to be extracted and can be used in diverse applications. Exploring applications in other high-value materials, such as adhesives, will not only enhance the valorization potential of the EPS but also promote resource recovery. This study aimed to characterize a water-soluble fraction extracted from the EPS collected at the demonstration plant in the Netherlands based on its chemical composition (amino acids, sugar, and fatty acids) and propose a proof-of-concept for its use as an adhesive. This fraction comprises a mixture of biomolecules, such as proteins (26.6 ± 0.3%), sugars (21.8 ± 0.2%), and fatty acids (0.9%). The water-soluble fraction exhibited shear strength reaching 36–51 kPa across a pH range of 2–10 without additional chemical treatment, suggesting a potential application as an adhesive. The findings from this study provide insights into the concept of resource recovery and the valorization of excess sludge at WWTPs.

## 1 Introduction

The paradigm surrounding wastewater has evolved from viewing it as a mere waste product to recognizing it as a valuable resource. There is a growing interest in transforming wastewater treatment plants (WWTPs) into resource recovery plants.

One example of the advancement in this field is the “Nereda technology” for wastewater treatment, which is based on a full-scale aerobic granular sludge (AGS) technology. The AGS technology offers numerous advantages over conventional activated sludge processes, such as a 75% reduction in footprint, a 30% reduction in energy, and lower operating costs (Pronk et al., 2015; Nancharaiah and Kiran Kumar Reddy, 2018). Additionally, the application of AGS technology has created new opportunities for resource recovery. In AGS, extracellular polymeric substances (EPS) produced by microorganisms form a hydrogel matrix, which is a dense network that gives the granular microbial structures their physical and chemical stability (Kehrein et al., 2020). These substances can be extracted and used for various high-value purposes while reducing waste sludge disposal and processing (Sam and Dulekgurgen, 2016; Bahgat et al., 2023). The total Dutch production of Kaumera is expected to reach up to 85,000 tons per year by 2030 (van Leeuwen et al., 2018).

The Netherlands has the world’s first two demonstration-scale installations (Zutphen and Epe WWTPs) to extract EPS from Nereda^®^ excess sludge granules under the product name Kaumera^®^. Kaumera extraction can account for 25–30% less removal and processing of waste sludge while recovering a versatile biopolymer (Kaumera, 2022). The EPS extraction process involves elevated temperature and pH condition to dissolve the biofilm matrix, which contains the target structural EPS (Felz et al., 2016). Thereafter, the solubilized EPS is precipitated out at an acidic pH and collected as the product named Kaumera. The recovered EPS primarily contains proteins and glycans (including polysaccharides and glycoconjugates) (Chen et al., 2023). Currently, some high-value applications for Kaumera^®^ include binders for composites, bio-stimulants in the agricultural and horticulture industry (van Leeuwen et al., 2018), flame-retardant coating (Kim et al., 2020), and bioadsorbant (Feng et al., 2021). Finding novel applications for Kaumera can further accommodate a circular economy.

A possible high-value EPS-based application could be a bio-based adhesive. Currently, adhesives worldwide are predominantly petroleum based. The prevalent use of the toxic substance formaldehyde as an additive, particularly in the wood industry, serves as an additional reason to explore non-toxic alternatives (Heinrich, 2019; Arias et al., 2021). As a result, bio-based adhesives produced from soy and starch have gained more recognition (Arias et al., 2021). Soy protein-based adhesives have exhibited promising adhesive properties in wood bonding. However, a drawback is the requirement for extensive chemical and physical treatment to obtain the desired properties (Arias et al., 2021). A similar case holds for starch. The stability of the adhesive is poor due to the polarity of starch; thus, additives and chemical modifications are required to produce an adhesive that is competitive with petrochemical adhesives (Heinrich, 2019; Arias et al., 2021). In addition, as both raw materials originate from food sources, it becomes uncertain whether the production of soy- and starch-based adhesives over the long term will be sufficient to cover the demand without impacting the primary food supply. Biopolymers produced from the wastewater treatment process, such as EPS (Kaumera), could provide a promising alternative for the current bio-based adhesive market. In addition, from a circular economy perspective, using EPS extracted from waste sludge as an adhesive can enhance the sustainability and economic viability of the wastewater treatment facility.

The objective of this study is to evaluate the water-soluble adhesive fraction of the extracted EPS (Kaumera) from a full-scale AGS wastewater treatment facility that treats industrial wastewater (Zutphen). To limit the chemical modifications of the Kaumera as a product, tap water was used to extract the water-soluble fraction. Chemical analysis of the sugar monomer, amino acid, and fatty acid composition was conducted, and the fraction was tested for its adhesive properties as a proof-of-concept. The information generated in this study will shed light on the concept of resource recovery and valorization of the excess sludge at WWTPs.

## 2 Materials and methods

### 2.1 The extraction of EPS (Kaumera) and the water-soluble fraction

**Figure 1:**
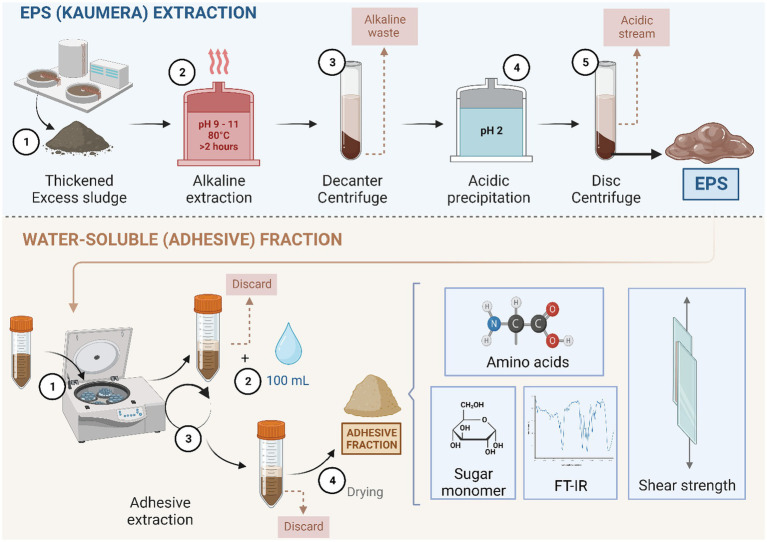
Schematic overview of the EPS produced from excess granular sludge at the Kaumera demonstration plant (Zutphen, the Netherlands) (as described in detail by Bahgat et al. (2023)) and the subsequent lab extraction of the water-soluble adhesive fraction and its analysis.

AGS was collected from full-scale WWTPs (Zutphen) in the Netherlands, which is operated with the Nereda® technology developed by Royal HaskoningDHV, the Netherlands. The extraction of EPS from aerobic granules was based on the alkaline heat extraction method described in detail by Bahgat et al. (2023). The extraction was performed within the pH range of 9 to 11 with the addition of 25% KOH at 80°C for 2 h. The EPS was precipitated followed by acidification with 30% HCl to the pH range of 2 to 4 (Figure 1).

To extract the water-soluble fraction, a 340-g extracted EPS was centrifuged for 15 min at 8671 x *g*. The supernatant was decanted. Tap water (100 mL) was added to the centrifuge bottle and centrifuged for 15 min at 8671 x *g* [based on the maximum capacity of 9,000 x*g* of the disk centrifuge in the Kaumera extraction plant (STOWA, 2019)]. The supernatant was frozen at −80°C, lyophilized, and stored at room temperature until further analysis (Figure 1). The volatile suspended solids (VS) and total suspended solids were measured according to the protocol of the American Public Health Association (APHA, 1998).

### 2.2 Characterization of the water-soluble fraction

#### 2.2.1 Functional group analysis by Fourier transform infrared spectroscopy

To identify the functional groups, the Fourier transform infrared (FTIR) spectrum of the lyophilized adhesive fraction was recorded using an FTIR spectrophotometer (Perkin Elmer, Shelton, United States) in attenuated reflectance (ATR) mode at room temperature with a wavenumber range from 550 cm^−1^ to 4,000 cm^−1^.

#### 2.2.2 Total carbohydrate and protein content

The lyophilized water-soluble fraction was dissolved in 1 mL of Milli-Q water with a concentration of 5 mg/mL. The total protein content was determined by the BCA protein assay following the manufacturer’s instructions with bovine serum albumin as a standard (Pierce™ BCA protein assay Kit, Thermo Scientific, USA). Protein absorbance was measured in duplicates at 562 nm using a multimode plate reader (TECAN Infinite M200 PRO, Männedorf, Switzerland). The total carbohydrate content of the EPS solutions, after five times dilution (1 mg/mL), was determined by the phenol–sulfuric acid method with glucose as a standard. The carbohydrate absorbance measurements were taken in cuvettes at 490 nm in duplicate with a VIS-spectrophotometer (HACH DR3900, Ames, IA, United States).

#### 2.2.3 Glycosyl composition and the fatty acid residue analysis using the TMS method

The glycosyl composition analysis of the water-soluble fraction was conducted at the Complex Carbohydrate Research Center (CCRC, the University of Georgia) by combined GC/MS of the O-trimethylsilyl (TMS) derivatives of the monosaccharide methyl glycosides produced from the sample by acidic methanolysis. These procedures were conducted as previously described by Santander et al. (2013). In brief, lyophilized EPS aliquots of 300 μg were added to separate tubes with 20 μg inositol as the internal standard. Methyl glycosides were then prepared from the dry sample following the mild acid treatment by methanolysis in 1 M HCl in methanol at 80°C for 16 h. The solution was then partitioned between hexane (1 mL) and deionized water (0.5 mL). The hexane phase was evaporated under a gentle N_2_ stream. The hexane phase was used to detect fatty acid residues. The samples in the water phase were re-N-acetylated with 10 drops of methanol, 5 drops of pyridine, and 5 drops of acetic anhydride and kept at room temperature for 30 min (for the detection of amino sugars). The sample was then per-o-trimethylsilyated by treatment with Tri-Sil (Pierce) at 80°C for 30 min. These procedures were conducted as described by Merkle and Poppe (1994). The GC/MS analysis of the per-o-TMS methyl glycosides and fatty acid residue was conducted on an AT 7890A gas chromatograph interfaced to a 5975B MSD mass spectrometer, using a Supelco EC-1 fused silica capillary column (30 m × 0.25 mm internal diameter). After the mass of each sugar monomer was determined using the GC/MS analysis, it was divided by the molecular mass to calculate the number of moles. Then, the molar ratio of each sugar monomer was calculated as each sugar monomer in mole/ total amount in mole.

#### 2.2.4 Free organic acid and amino acid analysis using NMR and GC/MS

To analyze the free organic acids and amino acids, nucleic magnetic resonance (NMR) and gas chromatography coupled with mass spectrometry (GC/MS) were performed. The adhesive fraction was dissolved in 2 mL of Milli-Q water with a concentration of 3–6 mg/mL using centrifuge filtration with Vivaspin® 6, 5 K MWCO (Sartorius, Gottingen, Germany). The eluted fraction was collected and centrifuged for NMR and GC/MS analysis.

For the detection of free organic acids and amino acids, NMR was employed according to the protocol described by Kim and Choi (2010).

For the analysis of amino acids with GC/MS, 100 μL of the eluted fraction was transferred to a glass vial containing 30 μL of 100 mg/mL NaCl and then lyophilized overnight. For the derivatization, 75 μL of acetonitrile and 75 μL of *N*-methyl-*N*-(*tert-*butyldimethylsilyl) trifluoroacetamide (MTBSTFA, Thermo Scientific) were added to the vial and incubated at 70°C for 1 h. The sample was centrifuged (2 min, 10,000 rpm, 4°C), and 80 μL of supernatant was transferred to an insert in the GC vial. The sample was analyzed by GC/MS using a 7890A GC (Agilent, Santa Clara, CA, United States) coupled with a 5975C MSD single quadrupole mass spectrometer (Agilent, Santa Clara, CA, United States) using a Zebron ZB-50 column (30 m × 250 μm internal diameter, 0.25 μm film thickness; Phenomenex, Torrance, CA, United States). The GC/MS method is described in detail by de Jonge et al. (2011).

### 2.3 Testing of the shear strength

**Figure 2:**
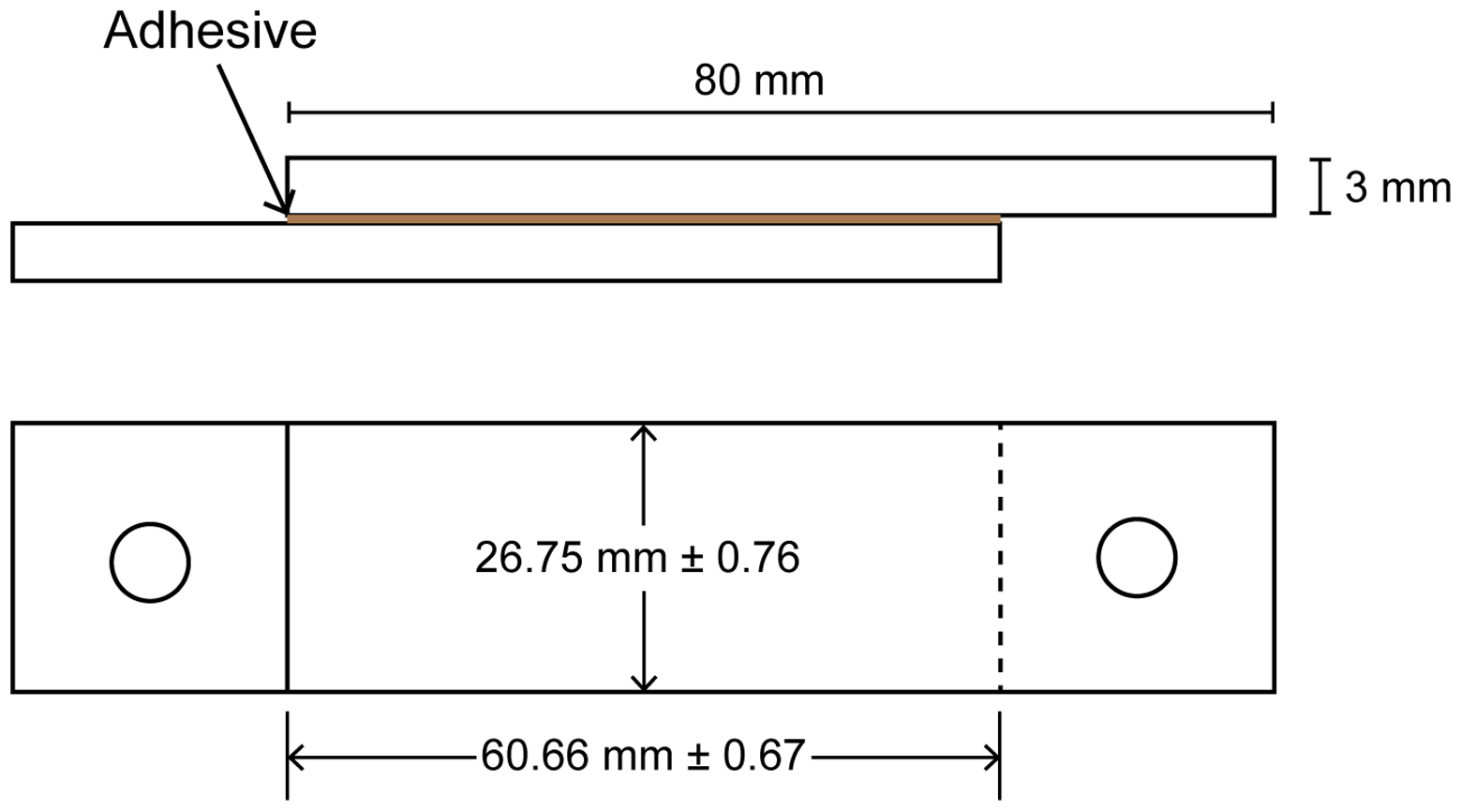
Detailed measurements of the specimens for the shear strength test on the side view (top) and top view (bottom).

To determine the shear strength of the water-soluble fraction, the lyophilized sample was dissolved in Milli-Q water with a concentration of 50% TS (w/v). KOH was added to reach different pH values of 2 to 10. The solution was further diluted with Milli-Q water to make a 30% w/v solution and vortexed vigorously. The final pH of each solution was measured. A volume of 100 μL of each pH solution was added on a polymethylmethacrylate (PMMA) slide with an approximate area of 16 cm^2^ (Figure 2). Another PMMA slide was used to spread the solution evenly while preventing the formation of bubbles. A constant pressure of 0.342 kPa was applied per slide while drying at room temperature (20°C) for 7 days. The area of the adhesive was measured at three points for each specimen, and the average area was noted and used for calculating the breaking force (Figure 2). The shear strength test was conducted using a universal tensile machine (Zwick RetroLine, ZwickRoell GmbH & Co. KG, Germany) with a loading speed of 10 mm/min. Both 10 N and 100 N load cells (Xforce HP, ZwickRoell GmbH & Co. KG, Germany) were used to measure the breaking force for the extracted fractions (pH of 2 to 10) of EPS and commercial adhesive (tesa^®^ Multi-Purpose Adhesive ecoLogo^®^), respectively. Using metal hooks and wire, the PMMA specimens were attached to the load cell and the movable cross-beam. The force needed at break was reported. All samples were tested in duplicate.

### 2.4 Morphology of the surface with the adhesive fraction

The PMMA slides after fracturing were visualized under a stereo zoom microscope (M205 FA, Leica Microsystems, Germany) connected to the Eert Vision Auto Focus Microscope camera (Eert Vision, the Netherlands), and the images were acquired using Eert C304 software (V1.0, Eert Vision, the Netherlands).

## 3 Results

### 3.1 The yield of the EPS and the water-soluble fraction

The yield of the EPS produced from the Kaumera demonstration plant at Zutphen was 22.0 ± 1.7% of the VS content of the sludge. The EPS itself consists of approximately 85% organic matter, while the yield of the water-soluble fraction is 6.2 ± 0.5% of the total weight of the EPS, which is 1.5% of the total organic content of the sludge. The *VS*/TS ratio of this fraction was 55.1 ± 3.4%. The absolute values of this fraction are shown in Supplementary Table S1.

### 3.2 Characterization of the water-soluble fraction

**Figure 3:**
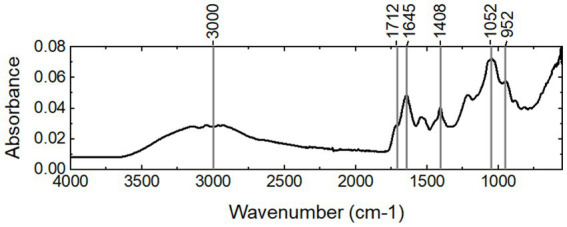
FTIR spectrum of the extracted water-soluble fraction.

To study the functional groups in the fraction, the FTIR spectrum of the sample was collected (Figure 3). First, the spectrum shows the typical bands of carboxylic acids: a strong, wide band in the region of 3,300–2,500 cm^−1^, centered at approximately 3,000 cm^−1^ for the O-H stretch, bands at 1712 cm^−1^ and 1,408 cm^−1^ for the C=O stretch and C–O stretch, respectively, and a band at 952 cm^−1^ for the O–H stretch. This aspect strongly indicates that there are carboxylic acids in the sample. Second, the band at approximately 1,645 cm^−1^ indicates that there are proteins with a random coil secondary structure. The band at approximately 1,052 cm^−1^ implies that there are carbohydrates.

The amount of proteins and carbohydrates was quantified using colorimetric methods. The total protein and total carbohydrate contents of the water-soluble fraction were 0.266 ± 0.003 mg protein/mg *VS* and 0.218 ± 0.002 mg carbohydrates/ mg *VS*, respectively. The sugar monomer composition is listed in Table 1. Glucose and rhamnose were the two relatively abundant sugar monomers. In addition, ribose, arabinose, rhamnose, fucose, xylose, mannose, and galactose were present. The fatty acid residues were determined using the GC/MS analysis after acidic methanolysis at the same time as the glycosyl composition. As shown in Table 2, diverse fatty acid residues were observed in the sample. The total mass was approximately 0.9% of the mass of the water-soluble fraction. Interestingly, the residues have a similar chain length to the fatty acids of lipid A reported in the literature (Lodowska et al., 2007). The primary fatty acids included C14:0, C16:0, C16:1, and C18:1, implying that lipopolysaccharides may be present in the water-soluble fraction. The strong signal of carboxylic acids in the FTIR spectrum may be due to the presence of fatty acid residues.

**Table 1:** Sugar monomer composition (molar ratio over the total amount in moles) in the water-soluble fraction of EPS produced at the Kaumera demonstration plant (Zutphen, the Netherlands).

Glycosyl residue	Ribose	Arabinose	Rhamnose	Fucose	Xylose	Mannose	Galactose	Glucose	N-acetyl glucosamine	Total (mass percentage in the sample (%))
Molar ratio	0.6	1.2	11.8	0.8	0.9	2.2	4.6	76.9	1.7	11.8

**Table 2:** Fatty acid residues were detected in the water-soluble fraction of EPS produced at the Kaumera demonstration plant (Zutphen, the Netherlands).

Fatty acid residue	6:0 (3-OH)	14:0	15:0	16:1	16:0	18:1	16:0 (3-OH)
Molar ratio	9.0	3.9	6.1	12.9	51.9	12.8	3.3

To detect the presence of free organic acids and amino acids in the sample, NMR and GC/MS were performed. While organic acids were hardly detected, diverse hydrophobic amino acids were found in the sample through NMR and GC/MS, including alanine, leucine, isoleucine, valine, methionine, phenylalanine, threonine, and tyrosine.

### 3.3 Evaluation of the shear strength

**Figure 4:**
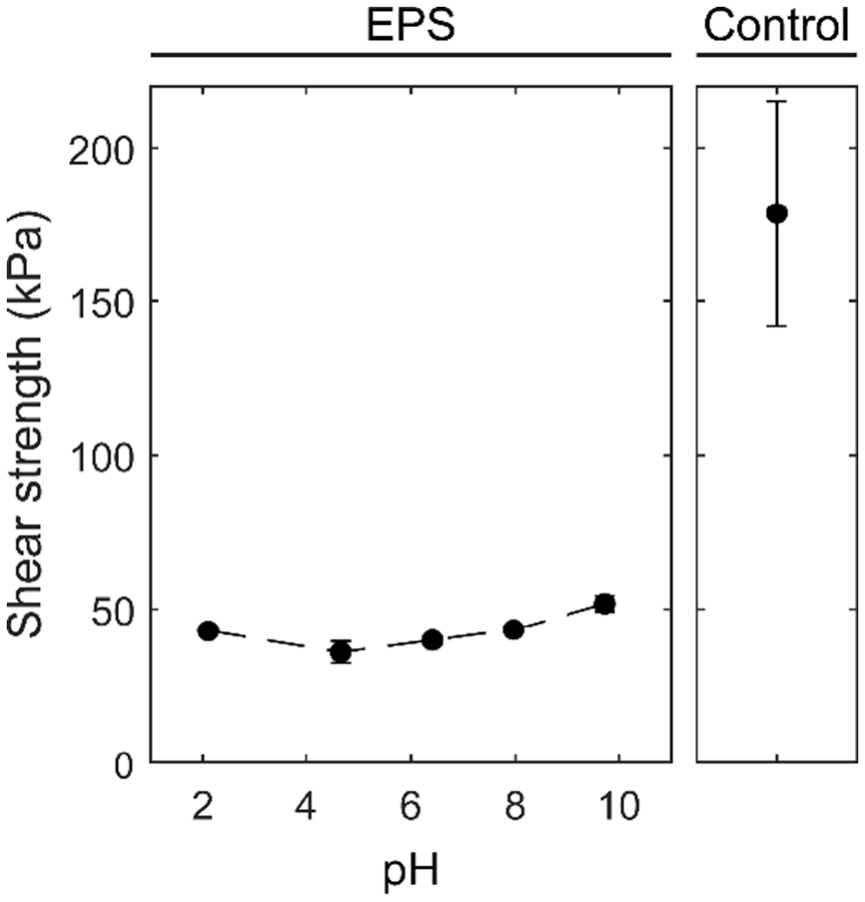
Influence of pH on the shear strength of the adhesive EPS fraction and the control, a water-based acrylic all-purpose glue (tesa® Multi-Purpose Adhesive ecoLogo®) on PMMA plates.

To evaluate the behavior of an adhesive, concentrated solutions (30% w/v) of the water-soluble fraction at different pH values were employed to glue two PMMA slides together. After curing, the shear strength was determined. As a comparison, a commercial water-based acrylic multipurpose glue (tesa^®^ Multi-Purpose Adhesive ecoLogo^®^) was tested. The pull-off force experiments on the two PMMA slides demonstrated that a certain amount of force was needed to pull off the two PMMA slides, which were glued together by the fraction extracted from the EPS. Upon breaking, the adhesive remained on both sides of the plates, showing a clear indication of cohesive failure (ASTM, 2010), while the commercial glue showed adhesive failure. The EPS adhesive fraction maintained its properties in a broad pH range, as demonstrated by the shear strength ranging from 36 to 51 kPa within the pH range of 2.1 to 9.7 (Figure 4). An increase in pH resulted in a 25% increase in shear strength. Compared to the commercial glue, the shear strength of the adhesive fraction is approximately one-fourth of that of the tesa® glue.

### 3.4 Morphology of the interface with the adhesive fraction

**Figure 5:**
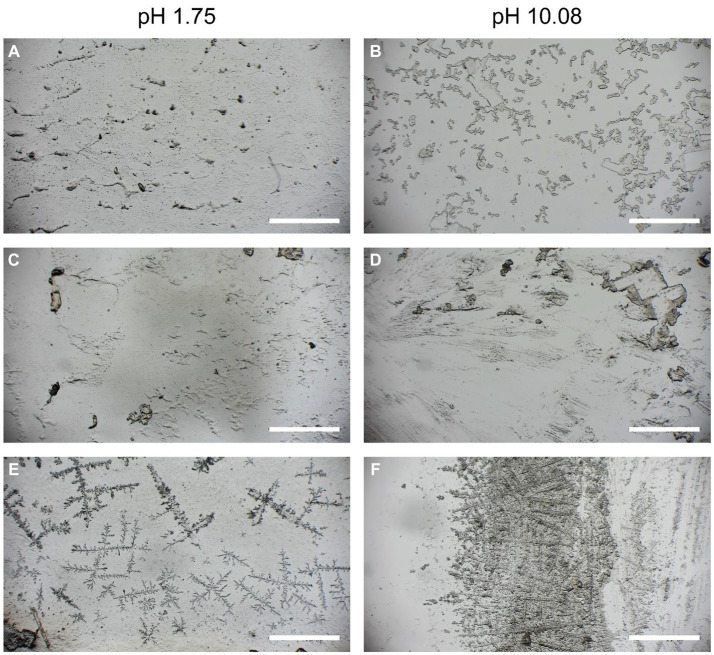
The adhesive on the surface of the PMMA slides after the shear strength breaking tests **(A–D)** and air-dried **(E,F)**.

After the shear strength tests of the PMMA plates, the morphology of the adhesive after breaking was visualized under a stereo zoom microscope. Clear differences in the pH range between 1.75 and 10.08 could be observed (Figure 5). For the pH value of 1.75, the interface with the adhesive fraction (Figures 5A,C) appeared more uniform, and the fractured surface showed a dense structure. However, for the pH value of 10.08, the adhesive interface appeared brittle, rough, and loose (Figures 5B,D), suggesting that the cohesive forces may have been different at the abovementioned pH values. When comparing the air-dried adhesives (Figures 5E,F), both pH values of 1.75 and 10.08 formed branches on the surface, while at the pH value of 10.08, the branched structure was denser than that at the pH value of 1.75. Salt crystals likely potassium chloride or sodium chloride from the extraction (Section 2.1) and based on the X-ray diffraction measurement (Supplementary Figure S1) were found precipitated on the branched structure.

## 4 Discussion

In the current research, a water-soluble fraction was extracted from the EPS produced at a resource recovery demonstration plant in the Netherlands. This fraction contained a mixture of biomolecules, such as proteins, free amino acids, sugars, and glycoconjugates (including lipopolysaccharides), and it formed a branched structure at the interface, holding the two surfaces together. Based on the pull-off test, the adhesive property was maintained at a broad pH range, with a shear strength of 36–51 kPa across a pH range of 2 to 10. The behavior of the adhesive fraction resembled that of a resin, suggesting that once it hardened, its adhesive property significantly increased. In comparison, some commercial adhesives, such as tesa® Multi-Purpose Adhesive ecoLogo®, which was included in the current research, have a pull-off strength of 175 kPa, while Tanglefoot®, an adhesive marketed for trapping insects, has a pull-off strength of 40–50 kPa (Eisner and Aneshansley, 1983). Thus, the pull-off strength of the EPS fraction falls within a similar range as that of commercial adhesives. On the other hand, lower pull-off strength does not necessarily preclude its use as an adhesive, as the strength requirements for adhesives vary depending on their intended application. The underlying mechanism of the adhesive property of the EPS fraction remains unknown. Interestingly, reported studies suggested that some proteins, glycoconjugates (e.g., glycoproteins and lipopolysaccharides), and amino acids can be used as adhesives (Abu-Lail and Camesano, 2003; Willett et al., 2005; Patel et al., 2013; Voigt et al., 2020; Heinritz et al., 2023).

The components observed in the adhesive fraction of the extracted EPS (Kaumera) might be used as a guide for identifying potential applications. For wood, paper, and carton adhesion, bio-based adhesives are frequently searched for as a sustainable alternative to petroleum-based adhesives (Heinrich, 2019). Studies reported that soy-protein and glucose-based adhesives showed a high bonding strength to plywood (Chen et al., 2013). As there are relatively high amounts of glucose and proteins in the adhesion fraction, its application for wood adhesion might be a good starting point to explore. In fact, more recently, activated sludge extract produced with deep eutectic solvent showed strong wood adhesion strength when mixed with a 1:1 ratio with glycerol triglycidyl ether (Xu et al., 2023). If the adhesive fraction of the extracted EPS from AGS already has adhesive properties, the addition of other chemicals may not be necessary. Another interesting component is the fatty acid. An adhesive prepared from soybean fatty acids has exhibited good pressure-sensitive adhesive properties that can be tailored for tapes and labels (Li and Li, 2014), implying that the application of tapes and labels might be another area to test in the future. One aspect to point out is the presence of a significant fraction of salts in the adhesion fraction, originating from the extraction (KOH and HCl, resulting in KCl). Whether this fraction enhances or decreases the adhesion property requires consideration.

Overall, the finding of these components in the adhesive fraction shows that it can be of interest in various ways for the development of a bio-based adhesive. Further studies should be conducted to gain a better understanding of the adhesion. A good starting point would be some adhesive properties (pressure-sensitive adhesion), effects of the environment (moisture and temperature), different formulations (concentrations and addition of carboxylic acids), and the effect of salts.

It is worth pointing out that the majority of commercial adhesives, such as poly(vinyl acetate), epoxy, phenol-formaldehyde, and polyurethane, are based on non-renewable and depleting petrochemical resources. Furthermore, numerous adhesives consist of residual toxic chemicals, which are harmful to the health of living beings and the environment (Patel et al., 2013). Therefore, green adhesives are significantly needed for society. On the other hand, the aerobic sludge granulation technology is rapidly spreading, with more than 100 full-scale WWTPs built worldwide. Sufficient waste granular sludge can be provided. With the two Kaumera demonstration production plants, more than 800 tons of Kaumera can be produced per year in the Netherlands. With the current research, it has been demonstrated that one fraction of the extracted Kaumera EPS contains biomolecules that can be used as green adhesives and have the ability to join surfaces together. Therefore, the development of the application of the EPS recovered from the excess sludge will provide a great opportunity for resource recovery and valorization, reducing the dependence on fossil fuel-based products. Further research is needed to understand the mechanisms and influence factors for the adhesive property.

## 5 Conclusion

A water-soluble fraction can be extracted from the EPS of excess AGS from a full-scale plant. This fraction contained a mixture of biomolecules, such as proteins, free amino acids, sugars, and fatty acids. The water-soluble fraction consisted of 26.6 ± 0.3% of total protein, with identified amino acids, including Ala, Leu, Ile, Val, Met, Phe, Thr, and Tyr. The total sugars made up 21.8 ± 0.2% of the water-soluble fraction, predominantly consisting of glucose (76.9%) and rhamnose (11.8%). The fatty acids accounted for 0.9% of the mass fraction, including C14:0, C16:0, C16:1, and C18:1 fatty acids. The water-soluble fraction exhibits shear strength from 36 to 51 kPa across a broad pH range of 2 to 10, indicating a potential application as a bio-based adhesive. This study demonstrates and identifies the main components of the water-soluble fraction of EPS, suggesting a promising application for the valorization of bio-based polymers recovered from waste sludge.

## Data availability statement

The original contributions presented in the study are included in the article/Supplementary material, further inquiries can be directed to the corresponding author.

## Author contributions

LC: Conceptualization, Investigation, Methodology, Writing – original draft. ÖE: Investigation, Methodology, Writing – review & editing. YC: Investigation, Methodology, Writing – review & editing. MP: Supervision, Writing – review & editing. ML: Funding acquisition, Writing – review & editing. YL: Conceptualization, Investigation, Supervision, Writing – original draft.
