# Supplementary material for: The water-soluble fraction of extracellular polymeric substances from a resource recovery demonstration plant: characterization and potential application as an adhesive
Source: Front Microbiol. 2024 Feb 26;15:1331120. doi: 10.3389/fmicb.2024.1331120 (PMC10925790; doi:10.3389/fmicb.2024.1331120)
Supplement: Supplementary file 1 [file Data_Sheet_1.pdf]

Supplementary Material

Supplementary Table S1. Yields and %volatile solids of the EPS and the obtained acidic fractions

Supplementary Figure S1. XRD Spectrum of the water-soluble fraction of EPS (magenta). Reference peaks of KCl (red) and NaCl (blue) were added to show the overlap of the signal.

Yields of the water-soluble extraction from EPS (Kaumera)

These percentages were calculated from the following absolute values as presented in Supplementary Table S1.

Supplementary Table S1. Yields and %volatile solids of the EPS and the obtained acidic fractions

	Yield % VS	%VS	Grams total solids
Kaumera	22 *	85	29.5
Water soluble fraction	6.2	55.1	1.8

* The Kaumera yield is defined here as the yield on the VSS content of the raw sludge

X-ray diffraction of the water-soluble fraction

X-ray diffraction(XRD) was performed using a Bruker D8 Advanced diffractometer with Cu-Kα source (Cu radiation wavelength: $K\alpha_1(100)$ = 1.54060 Å, $K\alpha_2(50)$ = 1.54439 Å) and Lynxeye-XE-T position sensitive detector. A silicon wafer sample is used and the amount of samples are very less. Geometry radius: 250.0 mm. Power: 40 kV and 25 mA. The measurement was performed as followed: a step size of 0.01°, and a measuring time of 0.1 s/step were employed. Geometry: theta-theta geometry (often called Bragg-Brentano or focusing geometry). A motorised varied-divergent slit of 5 mm. The scan range of 2theta is from 5-90°. The data was analysed using the Bruker software DiffracSuite.EVA vs 5.1. The references for KCl (PDF 75-0296), NaCl (PDF 77-2064) was obtained from the database. The identified minerals consist of 81.3% KCl and 18.7% NaCl (**Supplementary Figure S1**).

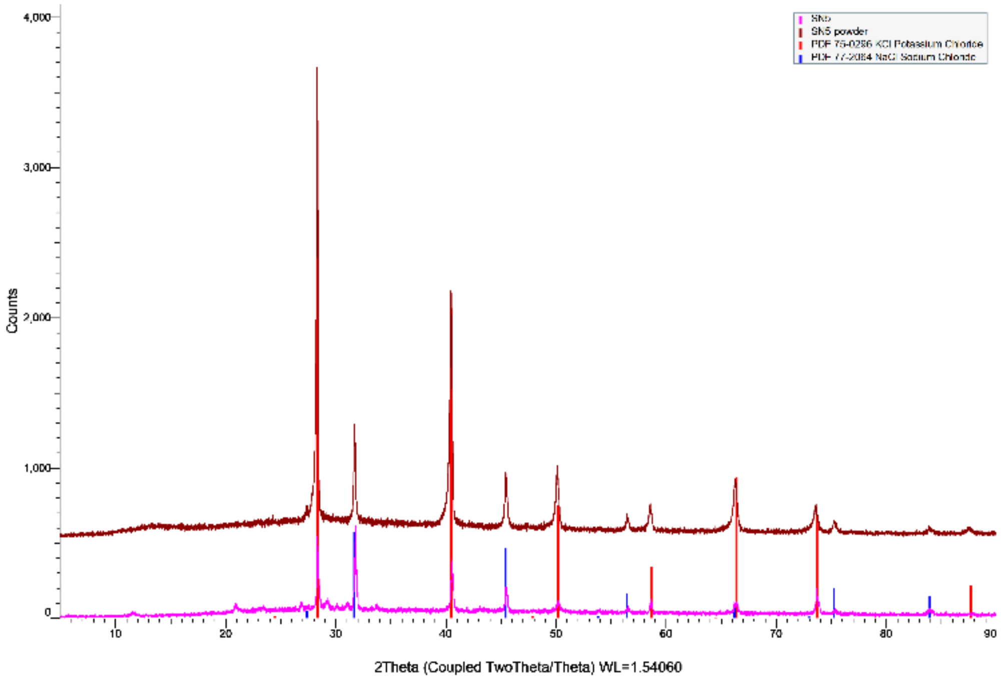

Supplementary Figure S1. XRD Spectrum of the water-soluble fraction of EPS (magenta and brown). Reference peaks of KCl (red) and NaCl (blue) were added to show the overlap of the signal.
